# Preformulation Studies of Ezetimibe-Simvastatin Solid Dispersions in the Development of Fixed-Dose Combinations

**DOI:** 10.3390/pharmaceutics14050912

**Published:** 2022-04-22

**Authors:** Agata Górniak, Adrianna Złocińska, Mateusz Trojan, Adrianna Pęcak, Bożena Karolewicz

**Affiliations:** 1 Laboratory of Elemental Analysis and Structural Research, Faculty of Pharmacy, Wroclaw Medical University, Borowska 211A, 50-556 Wroclaw, Poland; adrianna.zlocinska@umw.edu.pl (A.Z.); mftrojan@gmail.com (M.T.); adrianna121p@gmail.com (A.P.); 2 Department of Drug Form Technology, Faculty of Pharmacy, Wroclaw Medical University, Borowska 211A, 50-556 Wroclaw, Poland; bozena.karolewicz@umw.edu.pl

**Keywords:** simvastatin, ezetimibe, solid dispersion, eutectic, dissolution improvement, fixed-dose, dyslipidemia, cardiovascular disease

## Abstract

Two active pharmaceutical ingredients (APIs) with limited solubility, simvastatin and ezetimibe, prepared as a drug-drug solid dispersion (SD) was evaluated for physicochemical, microstructural, and aqueous dissolution properties. The simvastatin-ezetimibe SD was prepared using the co-grinding method in a wide range of weight fractions and differential scanning calorimetry (DSC) and X-ray powder diffraction (XRPD) were used to perform the phase composition analysis. DSC studies confirmed that simvastatin and ezetimibe form a simple eutectic phase equilibrium diagram. Analysis of Fourier transform infrared spectroscopy (FTIR) studies excluded strong interactions between the APIs. Our investigations have revealed that all studied dispersions are characterized by substantially improved ezetimibe dissolution regardless of simvastatin content, and are best when the composition oscillates near the eutectic point. Data obtained in our studies provide an opportunity for the development of well-formulated, ezetimibe-simvastatin fixed-dose combinations (for hypercholesterolemia treatment) with reduced ezetimibe dosages based on its dissolution improvement.

## 1. Introduction

Dyslipidemia or abnormal levels of lipids and lipoproteins in plasma is the most common major risk factor for cardiovascular disease (CVD) [1,2]. Dyslipidemia is a heterogeneous syndrome that can be distinguished into three clinical entities: hypercholesterolemia, atherogenic dyslipidemia, and hypertriglyceridemia [3]. Depending on the nature of the lipid disorders, various drugs with different mechanisms of action are used for treatment from the groups of available statins [4], fibrates [5], or cholesterol absorption inhibitors [6].

Statins are considered first-line drugs for the treatment of hypercholesterolemia and are the best-documented group of drugs in terms of effectiveness in primary and secondary CVD prevention [7]. Their mechanism of action is based on the competitive, specific, and reversible inhibition of hydroxymethylglutaryl-coenzyme A (HMG-CoA) reductase which is involved in the synthesis of endogenous cholesterol. Statins also lower triglyceride (TG) levels and increase the concentration of high-density lipoprotein cholesterol (HDL-C) [8].

In turn, ezetimibe (EZT) inhibits the absorption of exogenous cholesterol [9] by selectively blocking the NPC1L1 protein [10,11,12], however, its exact mechanism of action is not fully understood [11]. The inhibition of cholesterol absorption by EZT leads to the increased expression of low-density lipoprotein cholesterol (LDL-C) receptors, resulting in increased endogenous cholesterol uptake [13]. EZT use has been shown to be safe and well-tolerated—monotherapy is recommended for primary hypercholesterolemia in patients in whom statin therapy is contraindicated, as well as in patients with statin intolerance [14]. A significant reduction in cardiovascular events has been observed when adding EZT to statin therapy [15,16]. The introduction of this combination therapy within the last decade has allowed practitioners to achieve LDL-C targets in patients at high cardiovascular risk, refractory to statin monotherapy [17], and who experience side effects with high doses of statins [18,19]. The efficacy of the EZT and simvastatin (SIM) combination [20] has led to their approval for the treatment of primary [21] and secondary forms of hypercholesterolemia [22].

In recent years, fixed-dose preparations containing two or more active pharmaceutical ingredients (APIs) in a single pill have become more common in the pharmaceutical market [23]. A simplified therapy scheme with these preparations can be more patient-friendly [24,25] by increasing the likelihood of compliance with medical recommendations and consequently, improving the effectiveness of pharmacotherapy [26,27,28]. Fixed-dose combinations in the treatment of hyperlipidemia work to improve efficacy, provide a favorable safety profile, and complement the mechanism of action of each compound to provide enhanced hypolipidemic and pleiotropic effects [29,30]. For a decade, Inegy (Vytorin or Goltor), a product produced (Merck Sharp & Dohme and Schering Plough) in four EZT and SIM dose combinations (1:1, 1:2, 1:4, and 1:8, respectively) has been available on the market [31,32].

The Biopharmaceutical Classification System (BCS) classifies both EZT and SIM as class II drugs [33,34]. EZT is practically insoluble in aqueous media and the solubility of anhydrous and hydrated forms of the substance is approximately 12 µg/mL and 8 µg/mL, respectively [33]. The solubility of SIM in water is twice as high at approximately 30 µg/mL [35]. Both these APIs have low solubility and high permeability and exhibit dissolution rate-limited absorptions and high variability in pharmacological effects. Various strategies have been used to enhance the solubility, dissolution rate, and/or oral bioavailability of poorly soluble drugs. The most commonly used include the formation of salts and obtaining solid dispersions (SDs), co-amorphous systems, nanocrystals, or cocrystals [36].

Eutectic mixtures prepared with crystalline components are classified as first-generation SDs or crystalline SDs having a well-defined microstructure. Eutectic mixtures are not considered to be new chemical entities or new crystal forms, and thus do not require clinical trials [37,38]. The increased interest in the use of eutectic mixtures in the pharmaceutical industry is associated with their cost-effectiveness, ease of production and scaling up preparation methods, and higher stability in comparison to the amorphous materials [39,40]. Eutectic mixtures can be prepared using multiple different techniques such as the melting followed by cooling method, solvent evaporation technique, spray drying technique, electrospray deposition technique, and mechanical methods including neat co-grinding, ball milling, or liquid-assisted grinding [41,42]. The resulting formulation structure depends on the preparation method and the properties of its constituents. Despite interest in these systems, they are still under preliminary development with regard to pharmaceutical applications and have been relatively unexplored compared to salts and cocrystals [40,43,44].

Despite numerous studies on drug-drug eutectic mixtures [41,42] as an approach to enhancing component solubility and dissolution rates [44,45], there is still a lack of studies characterizing mixtures containing two poorly water-soluble or practically insoluble drugs. In view of the increasing demand for fixed-dose combination products containing poorly or practically insoluble drugs, this study aimed to evaluate the physicochemical properties of the SIM-EZT system, as well as examine the influence of observed eutectic mixtures on the dissolution rate of these poorly-soluble APIs.

## 2. Materials and Methods

### 2.1. Materials

**Figure 1:**
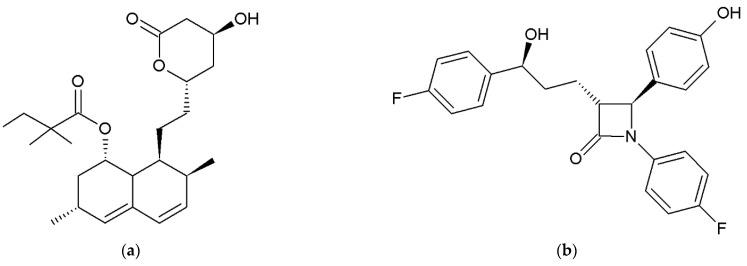
Chemical structures of simvastatin (**a**) and ezetimibe (**b**).

SIM (CAS 79902-63-9) for pharmaceutical application, with chemical formula [(1S,3R,7S,8S,8aR)-8-[2-[(2R,4R)-4-Hydroxy-6-oxotetrahydro-2H-pyran-2-yl]ethyl]-3,7-dimethyl-1,2,3,7,8,8a-hexahydro-1-naphthalenyl] 2,2-dimethylbutanoate and with structure provided in Figure 1a, was donated by Polpharma SA (Starogard Gdański, Poland). EZT (CAS 163222-33-1) for pharmaceutical application, with the chemical formula (3R,4S)-1-(4-fluorophenyl)-3-[(3S)-3-(4-fluorophenyl)-3-hydroxypropyl]-4-(4-hydroxyphenyl)azetidin-2-one and structure provided in Figure 1b, was donated by Pharmaceutical Research Institute (Warsaw, Poland). SIM was used as received and EZT was dehydrated by drying in an oven at 100 °C and by slow cooling to room temperature. Dehydrated EZT was stored in a desiccator until further use. An acetate buffer solution (pH 4.5) was obtained by diluting the suitable volume of concentrate (J.T.Baker, Deventer, The Netherlands) in deionized and degassed water. Acetonitrile (HPLC grade) was purchased from Merck (Darmstadt, Germany). Sodium lauryl sulfate (SLS) was purchased from Stanlab (Lublin, Poland).

### 2.2. Preparation of SIM-EZT Dispersions

The SIM-EZT dispersions were prepared using the co-grinding method. In this method, accurately weighed quantities of both APIs were thoroughly triturated for 15 minutes using a pestle and mortar to achieve a homogeneous mixture. Subsequently, each dispersion was placed into a sealed vial and preserved with a desiccator before use. The compositions of various dispersions are displayed in Table 1.

### 2.3. API Content Analysis

The content of APIs in each dispersion was determined using the high-performance liquid chromatography (HPLC) method. An Ultimate 3000 Dionex (Thermo Fisher Scientific, Waltham, MA, USA) apparatus, a Purospher RP-18 column (250 cm × 4 mm, 5 μm, Merck, Kenilworth, NJ, USA), and an isocratic elution with a flow rate of 1 mL/min have been applied. The ingredients were eluted with a mobile phase consisting of a phosphate buffer (pH 4.5): acetonitrile mixture, 35:65 (*v*:*v*), and identified by a UV-VIS DAD detector at 238 nm. The retention time was 3.6 min and 12.9 min for EZT and SIM, respectively. External standards obtained in the range of 5–50 μg/mL were used to plot calibration curves for SIM and EZT (linearity r^2^ = 0.999). The solutions for API content analysis were prepared by dissolving 10 mg of each obtained SD in 100 mL of the mobile phase. Next, the solutions were filtered through a 0.45 µm pore size membrane filter and quantified by HPLC.

### 2.4. Differential Scanning Calorimetry (DSC) Measurements

The thermal characteristic of SIM, EZT, and their binary dispersions were evaluated using a DSC Polyma 214 (Netzsch, Selb, Germany) heat-flux type differential scanning calorimeter. The apparatus was calibrated for temperature and enthalpy using indium (156.6 °C), tin (231.9 °C), bismuth (271.4 °C), and zinc (419.5 °C) standards. The investigated samples (4–5 mg), were weighed in standard aluminum crucibles (25 μL) and sealed with a pierced lid. An identical empty crucible was used as a reference. The DSC heating curves were obtained within the temperature range of 25 °C to 190 °C at a constant heating rate of 5 °/min. Dry nitrogen (purity 99.999%) with a flow rate of 25 mL/min was used as a protective gas.

### 2.5. Fourier Transform Infrared (FTIR) Spectroscopy

The FTIR spectra were registered by means of Nicolet iS50 FTIR (Thermo Scientific, Waltham, MA, USA) Spectrometer equipped with an Attenuated Total Reflection (ATR) module with an embedded diamond crystal. Each spectrum was defined as an average of 32 scans, registered in the wavenumber range of 400 to 4000 cm^−1^ with a resolution of 4 cm^−1^. All obtained spectra were normalized and the baseline was corrected. The OMNIC software version 5.0 (Thermo Scientific, Waltham, MA, USA) was used for the analysis of the registered spectra.

### 2.6. X-ray Powder Diffractometry (XRPD)

X-ray diffraction studies were carried out using a D2 Phaser (Bruker AXS, Karlsruhe, Germany) apparatus equipped with a horizontal goniometer operating in the 2Theta mode with a one-dimensional LYNXEYE^®^ detector. The X-ray source was CuKα radiation at a current of 10 mA and a voltage of 30 kV. The diffractometer was calibrated with a corundum standard provided by Bruker AXS. A low-background holder was used to obtain patterns of all examined samples. Studies were performed at room temperature over a 2θ range of 5° to 35° with a step size of 0.02° and a 1 s irradiation time per step. During the test, the samples were rotated at a speed of 15 rpm. The obtained XRPD patterns were evaluated using Diffrac.Eva V 3.2 software (Bruker AXS, Karlsruhe, Germany).

### 2.7. Scanning Electron Microscopy (SEM) Imaging

SEM was used to observe the morphology of the pure APIs and their binary dispersions. The SEM images were obtained by means of a field emission scanning electron microscope (Zeiss, Jena, Germany, Sigma 500 VP). To improve the discharge process, the samples were coated with gold prior to the experiments using a Quorum machine (Quorum International, Fort Worth, TX, USA). The sputter parameters were as follows: current 40 mA, time 50 s.

### 2.8. In Vitro Dissolution Testing

In vitro dissolution testing for SIM, EZT, and its binary SDs was carried out in triplicate form for each sample using a USP type 2 (rotating paddle) apparatus VK 7025 (Varian Inc., Palo Alto, CA, USA). Experiments were conducted at 37 ± 0.5 °C with a paddle rotation of 50 rpm over a 1.5 h time frame using powdered samples (50 mg) in 500 mL of 0.5% SLS aqueous solution. At time intervals of 5, 10, 20, 30, 40, 50, 60, and 90 min, samples of the solution (3 mL) were automatically taken by sampling cannulas equipped with a 45 μm cannula filter and analyzed using the HPLC method, described in Section 2.3. The sink conditions in dissolution tests can lead to an increase in the dissolution rate of examined formulations and may cause difficulties in the discrimination of dissolution profiles of different formulations, as well as not guaranteeing the absence of saturation effects. Since the aim of this work was to examine the reciprocal impact of two poorly soluble APIs on their release from binary solid dispersions, the dissolution studies were performed with the same sample mass of each SDs (50 mg) under non-sink conditions for EZT.

## 3. Results and Discussion

### 3.1. Drug Content

API content in the examined SDs was found to be in the range of 98.44% to 101.71% of the declared amount of EZT, and 97.44% to 101.74% of the declared amount of SIM. Results of the API content studies conducted on the examined dispersions are presented in Table 1.

### 3.2. Thermal Analysis and Phase Transformation Behaviour

**Figure 2:**
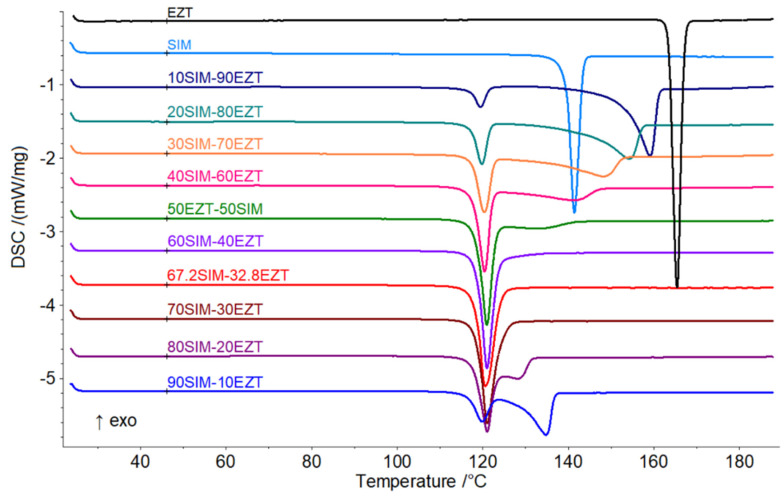
DSC heating curves of pure ingredients (SIM, EZT) and SIM-EZT SDs.

**Figure 3:**
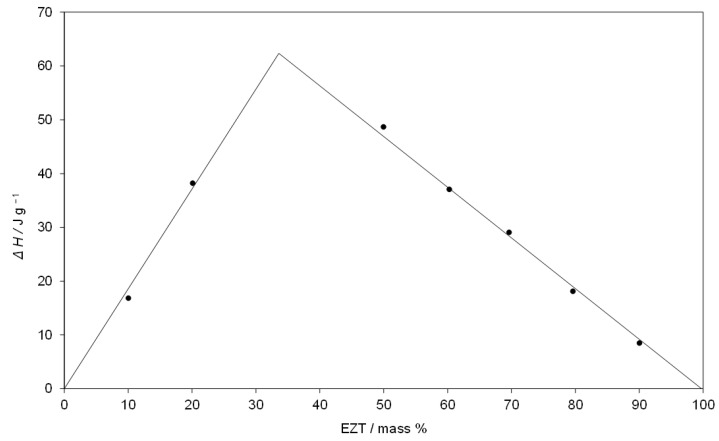
Tamman’s triangle of eutectic melting enthalpy, ΔH at 117.5 °C versus EZT mass %.

**Figure 4:**
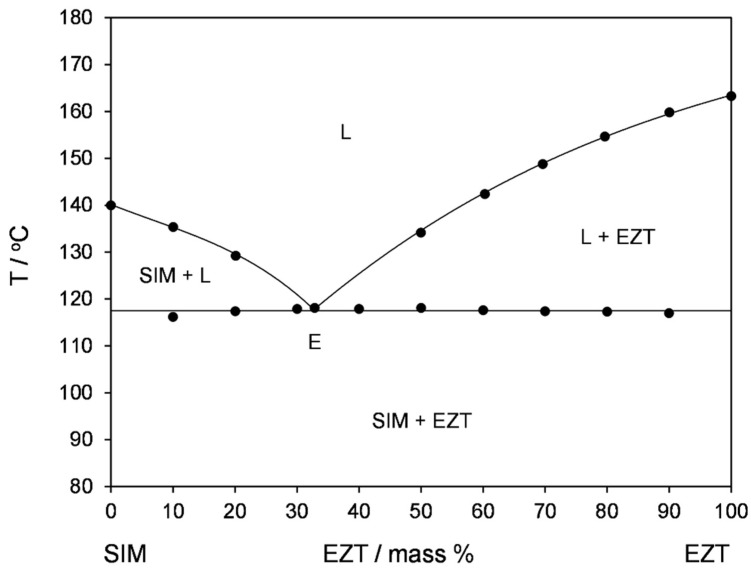
Phase diagram of SIM-EZT system.

The obtained SDs underwent thermal analysis to determine the proper eutectic composition. The phase diagram of the system SIM-EZT and Tamman’s triangle were constructed for this purpose. Knapik-Kowalczuk et al. [46] previously reported on the thermal properties of binary SIM-EZT physical mixtures to determine the ternary ezetimibe-simvastatin-fenofibrate system. This study found that SIM and EZT form a eutectic system, however, neither the eutectic reaction temperature values nor the dependence of eutectic enthalpy vs. mixture composition has been presented. On DSC curves (Figure 2), we observed a phase transition to occur at a constant temperature which is characteristic for eutectic systems. An invariant temperature of 117.5 °C was determined as the onset of phase transition and corresponded to the observed eutectic reaction: solid SIM + solid EZT = liquid (L). The enthalpy values of phase transitions detected during the heating of SIM-EZT samples (Figure 2) are presented in Table 2. Based on the analysis of the DSC experiments, we conclude that the SIM-EZT eutectic mixture contains 67.2 mass % of SIM and 32.8 mass % of EZT corresponding to a SIM:EZT mole ratio of 2:1. We determined the eutectic point composition to be the value of the abscissa at the maximum of Tamman’s triangle (Figure 3) [42]. The course of Tamman’s triangle confirms no reciprocal miscibility in the solid-state as the eutectic reaction’s enthalpy values fall to zero as the mixture composition approaches pure ingredients. Thus, we concluded that SIM and EZT form a simple eutectic system with the shape shown in Figure 4.

### 3.3. XRPD Studies

**Figure 5:**
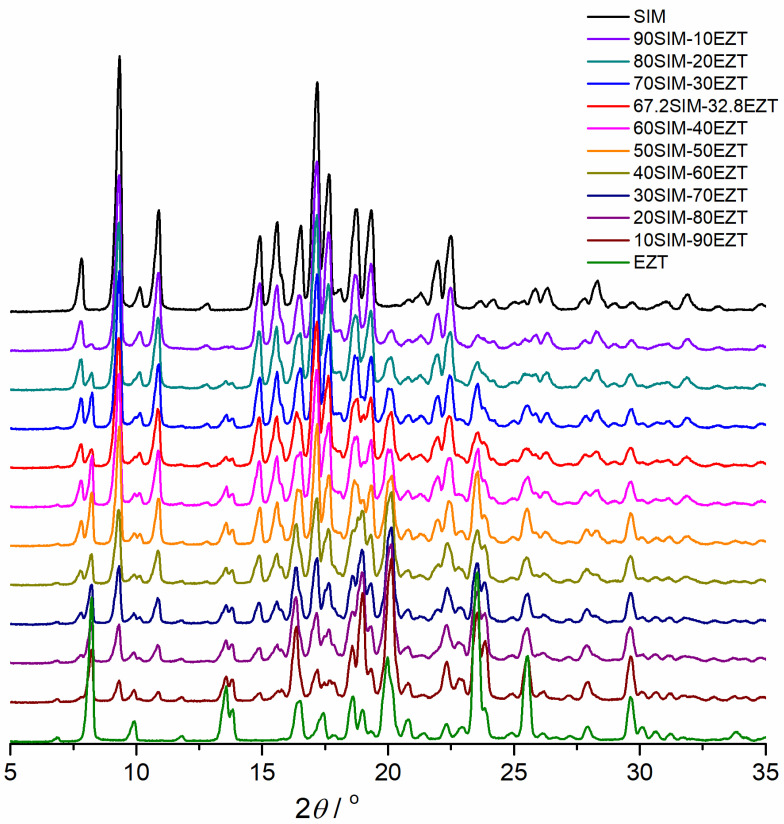
X-ray diffraction patterns of SIM, EZT, and obtained SIM-EZT SDs.

The SIM and EZT XRPD patterns and those received for SIM-EZT SDs are presented in Figure 5. A lot of distinct reflections indicating the crystalline nature of the investigated powders can be observed on the pure APIs and SDs patterns. The XRD reflections were visible on the SIM pattern at 2*θ* diffraction angles of 7.8°, 9.4°, 10.9°, 15.0°, 17.2°, and 22.5° and the EZT pattern at 8.2°, 13.6°, 13.8°, 16.4°, 18.6°, 19.0°, 20.1°, 22.3°, 23.5°, 25.5°, 28.0°, and 29.7° were also present on the analyzed SIM-EZT patterns at the same angular positions. On the XRPD patterns obtained for the SIM-EZT dispersions, only reflections distinctive for SIM and EZT were observed. This confirms that during the process of SDs preparation, no different crystal phase appeared and also excludes any chemical interactions between the SDs components in the solid state at room temperature. The presence of clearly outlined peaks confirms that during grinding the mixtures were not transformed into an amorphous state. Furthermore, the frictional force acting on the EZT and SIM particles during grinding did not cause any polymorphic transitions.

### 3.4. FTIR Spectroscopy Analysis

**Figure 6:**
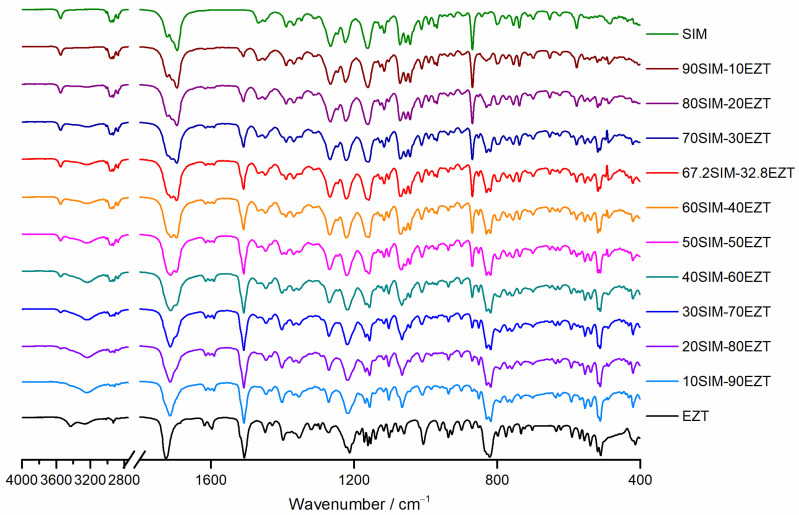
FTIR spectra of pure ingredients (SIM, EZT) and SIM-EZT SDs.

The FTIR spectra were registered and analyzed to evaluate for interactions occurring at the molecular level between EZT and SIM in the obtained drug-drug SDs. The FTIR spectra obtained for raw SIM, EZT, and their binary SDs are shown in Figure 6. SIM is characterized by distinctive bands at wavenumbers of 3547 cm^−1^ (free O–H stretching vibration), 2951 cm^−1^, 2930 cm^−1^, 2872 cm^−1^, 1467 cm^−1^ (aliphatic C–H vibrations), 1695 cm^−1^ (C=O ester vibration), 1266 cm^−1^, and 1162 cm^−1^ (C-O-C ester and lactone vibrations). Characteristic absorption bands have also been observed for raw EZT related to O–H stretching vibrations at 3430 cm^−1^ and 3265 cm^−1^, C=O β-lactam stretching vibration at 1725 cm^−1^, benzene ring C=C stretching vibration at 1507 cm^−1^, C–F stretching vibration at 1212 cm^−1^, C–O stretching vibration at 1063 cm^−1^, and para-substituted benzene ring vibration at 821 cm^−1^. These values correspond well with data reported in the literature for SIM [47] and EZT [45]. The comparative analysis of FTIR spectra collected for the studied SDs revealed absorption bands characteristic of their constituents. Apart from intensity changes, related to the difference in API content, no new bands indicating strong chemical interactions were observed. Moreover, neither meaningful shifting nor band broadening was detected indicating the absence of weak interactions (such as hydrogen bonds).

### 3.5. Shape and Surface Morphology

**Figure 7:**
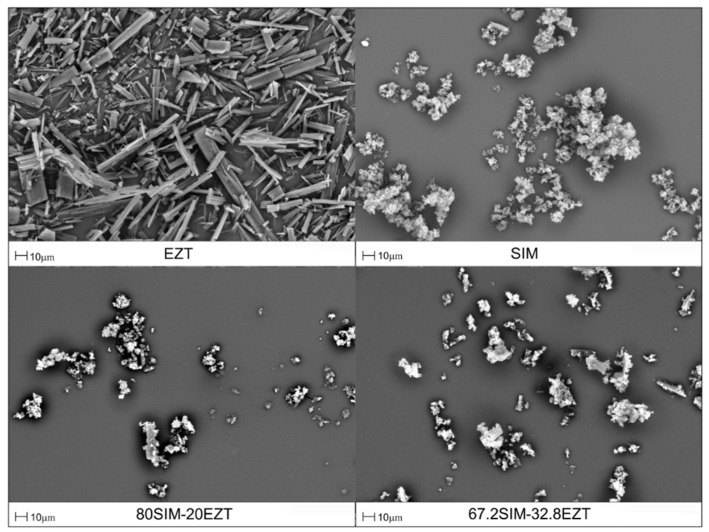
SEM photomicrographs of EZT, SIM, and selected SIM-EZT SDs.

SEM photomicrographs obtained of the pure drugs and selected samples of SIM-EZT SDs revealed a surface morphology of the investigated samples as shown in Figure 7. The SEM picture of SIM demonstrates a fine crystalline powder, whereas the SEM picture of EZT reveals characteristic needle-shaped crystals. The SEM pictures of selected SDs demonstrate the presence of irregular particles with a crystalline nature, which has also been confirmed in XRPD studies.

### 3.6. Dissolution Tests Analysis

**Figure 8:**
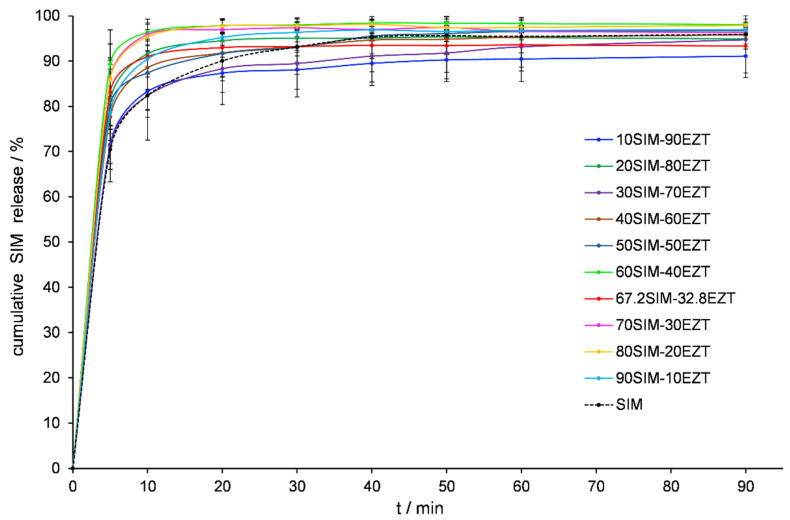
Dissolution profile of SIM released from SIM-EZT dispersions.

**Figure 9:**
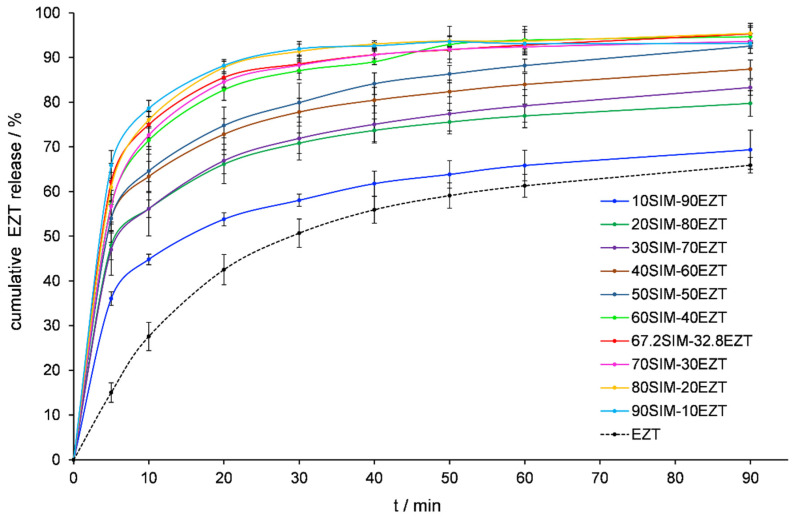
Dissolution profile of EZT released from SIM-EZT dispersions.

The bioavailability of the API after oral administration depends substantially on its solubility and dissolution rate in an aqueous environment, as well as on the permeation through gastrointestinal membranes [48]. Sufficient absorption is difficult to achieve when at least one of these factors is unfavorable. Insufficient water solubility is a parameter that affects the pharmaceutical availability of many active substances. EZT is practically insoluble in water. The aqueous solubility of EZT hydrated forms is estimated to be 0.008 mg/mL, whereas EZT anhydrous forms reach a solubility level of 0.012 mg/mL [33]. The dissolution profiles were evaluated for the APIs released from prepared SDs and presented in Figure 8 and Figure 9. Figure 9 shows an increase in EZT dissolution is achieved for all SDs and was substantially improved when SIM’s mass percentage oscillated near the eutectic composition point. These mixtures released twice the amount of EZT compared to raw API samples within the first 20 min of testing.

## 4. Discussion

The successful treatment of dyslipidemia has a very important meaning in the primary and secondary prevention of cardiovascular events [49,50]. The primary goal of dyslipidemia treatment is to lower LDL-C. A combination with EZT should be considered if the LDL-C target goal is not achieved despite using the maximum tolerable dose of a statin.

Our research shows that a eutectic formation of the binary drug-drug system of SIM-EZT significantly increases the dissolution of EZT compared to the pure drug. Numerous reports have described the impact of hydrogen bond formation or API amorphization [51,52,53] in enhancing the dissolution of SD preparations. Our thermal and spectroscopic studies excluded these influences as factors when SIM-EZT SDs were prepared by simple co-grinding. This study confirms the direct influence the formation of a co-grinding-mediated eutectic mixture can have on improving the dissolution profile of the studied dispersions.

There are a lot of methods that result in the formation of eutectics [42]. Most of them are costly and require the use of numerous operations or factors harmful to the environment such as organic solvents or the use of a lot of energy [42,54,55,56]. The use of the co-grinding method in SD preparation is very convenient, efficient, and easier to scale up than other methods based on cost absorption and the requirements for the use of non-ecological organic solvents.

Data obtained from our study illustrates a great opportunity in the development of well-formulated fixed-dose combinations for hypercholesterolemia treatment. This is particularly significant in the development of polypill preparations that are excellent for addressing adherence problems with CVD polypharmacy [27,28,57,58,59]. This approach has the added benefit of reducing the effective dose of API while also eliminating excipients, such as SLS, required in oral solid formulations as wetting agents for the purpose of increasing dissolution rates of poorly soluble APIs. The easily prepared, rapidly dissolving SIM-EZT SDs are a promising drug delivery system for the administering of both APIs in fixed doses with improved solubility in the aqueous environment of the gastrointestinal tract. However, in vivo studies of animal subjects are needed to analyze and confirm the real effect of dispersion compositions on the oral bioavailability of studied APIs, and their effectiveness in improving lipid profiles. The degree of micronization is an important aspect once a mechanical force has been used in the formation of an SD. Therefore, in future pre-formulation studies, it is necessary to investigate the effects of various grinding parameters (time, oscillations, applied force) on the grain size of the dispersion.

## 5. Conclusions

The formulation of SIM-EZT SDs forms a eutectic mixture suitable for improving the solubility and dissolution rates of EZT without affecting the solubility of SIM. This novel approach includes the advantages of a eutectic mixture characterized by improved aqueous dissolution properties during the formulation of oral polypills intended for fixed-dose dyslipidemia treatment. The SIM-EZT eutectic can be prepared by a simple co-grinding method, which is more convenient, efficient, and easier for production and scaling up than other methods based on high energy consumption or require the use of non-ecological organic solvents.

## Figures and Tables

**Table 1:** Compositions of investigated SIM-EZT dispersions and APIs average content.

Sample Code	Composition/Mass %		Average Content /%	
	Simvastatin	Ezetimibe	Simvastatin	Ezetimibe
90SIM-10EZT	90.0	10.0	101.74 ± 0.03	101.71 ± 0.15
80SIM-20EZT	80.0	20.0	97.44 ± 0.15	98.44 ± 0.15
70SIM-30EZT	70.0	30.0	99.39 ± 0.04	100.53 ± 0.05
67.2SIM-32.8EZT	67.2	32.8	99.39 ± 0.06	100.66 ± 0.05
60SIM-40EZT	60.0	40.0	98.61 ± 0.03	100.02 ± 0.06
50EZT-50SIM	50.0	50.0	98.17 ± 0.03	99.98 ± 0.06
40SIM-60EZT	40.0	60.0	98.86 ± 0.14	100.41 ± 0.21
30SIM-70EZT	30.0	70.0	98.78 ± 0.04	100.44 ± 0.19
20SIM-80EZT	20.0	80.0	97.76 ± 0.05	99.27 ± 0.04
10SIM-90EZT	10.0	90.0	97.56 ± 0.11	99.25 ± 0.06

Data expressed as mean ± SD (n = 3).

**Table 2:** Experimental temperatures and enthalpy values of phase transitions were observed in the SIM-EZT system.

Sample Code	Eutectic Invariant		Liquidus
	T/°C	ΔH/J g^−1^	/°C
SIM			140.0 ± 0.1
90SIM-10EZT	116.2 ± 0.2	16.8 ± 0.6	135.3 ± 0.2
80SIM-20EZT	117.4 ± 0.2	38.2 ± 1.3	129.2 ± 0.2
70SIM-30EZT	117.9 ± 0.2	-	
67.2SIM-32.8EZT	118.1 ± 0.3	-	
60-SIM-40EZT	117.9 ± 0.1	-	
50SIM-50EZT	118.1 ± 0.1	48.7 ± 1.0	134.2 ± 0.2
40SIM-60EZT	117.6 ± 0.2	37.1 ± 0.5	142.4 ± 0.1
30SIM-70EZT	116.9 ± 0.1	29.1 ± 0.2	148.8 ± 0.1
20SIM-80EZT	117.3 ± 0.3	18.1 ± 0.9	154.7 ± 0.1
10SIM-90EZT	117.0 ± 0.2	8.5 ± 0.1	159.8 ± 0.3
EZT			163.3 ± 0.1

Data expressed as mean ± SD (n = 3).

## Data Availability

Not applicable.
